# Predictive Value of SMART-COP for Adverse Outcomes in Older ICU Patients with Community-Acquired Pneumonia: A Comparison with CURB-65, SOFA, and APACHE II

**DOI:** 10.3390/jcm15145664

**Published:** 2026-07-19

**Authors:** Ayse Muge Karcioglu, Hatice Zeynep Atli, Ugurcan Degdas

**Affiliations:** 1 Intensive Care Unit, Etlik City Hospital, Ministry of Health, 06170 Ankara, Türkiye; drzeynepatli@gmail.com; 2 Department of Anesthesiology and Reanimation, Etlik City Hospital, Ministry of Health, 06170 Ankara, Türkiye; ugurcdegdas@gmail.com

**Keywords:** pneumonia, critical care, prediction score, geriatric, mortality

## Abstract

**Background/Objectives**: Community-acquired pneumonia (CAP) is a leading cause of ICU admission and death in older adults, yet the severity scores used in this setting were not developed for, or validated in, older intensive care patients. We compared a pneumonia-specific score (SMART-COP) with the general ICU scores SOFA and APACHE II and with CURB-65 for predicting adverse outcomes in this population. **Methods**: In this single-center prospective observational study, 227 consecutive patients aged ≥65 years admitted to the ICU with CAP were enrolled. SMART-COP and CURB-65 were calculated at hospital admission and SOFA and APACHE II at ICU admission. The discrimination of each score for four outcomes—in-ICU mortality, need for intubation, vasopressor requirement, and hospital-acquired pneumonia (HAP)—was assessed by ROC analysis, and areas under the curve (AUCs) were compared pairwise using the DeLong test. **Results**: All four scores predicted mortality, intubation, and vasopressor requirement (all *p* ≤ 0.001), but none discriminated well for HAP (all AUCs < 0.70). SMART-COP had the highest AUCs for mortality (0.846) and intubation (0.870), significantly exceeding CURB-65 (both *p* < 0.01) while performing comparably to SOFA and APACHE II. SOFA was the strongest predictor of vasopressor requirement (AUC: 0.867). **Conclusions**: In older ICU patients with CAP, SMART-COP—calculable from routine parameters at admission—matched far more complex ICU scores for mortality and intubation and outperformed CURB-65. The optimal score depends on the outcome of interest.

## 1. Introduction

Community-acquired pneumonia (CAP) is the leading infectious cause of adverse outcomes, including mortality, and is estimated to account for approximately 2.2 million deaths annually [1]. CAP results in approximately 1.4 million emergency department visits and 740,000 hospitalizations per year in the United States, and over 1 million hospital admissions annually in Europe, with direct healthcare expenditures exceeding 14 billion US dollars in the US and surpassing 10 billion euros in Europe [2,3,4,5]. Advanced age is a well-established risk factor for developing CAP, with incidence rates rising sharply in older populations—from approximately 300 per 100,000 in adults aged 18–64 years to over 2000 per 100,000 in those aged 65 and older [6,7]. Furthermore, advancing age has been identified as an independent predictor of adverse outcomes in patients with CAP, including intensive care unit (ICU) admission, in-hospital mortality, and overall mortality [1,8].

Accurate assessment of disease severity and determination of level of care are crucial for optimal management of CAP. Several severity scores have been developed to guide level-of-care decisions and predict adverse outcomes in CAP, including CURB-65, the Pneumonia Severity Index (PSI), and SMART-COP, each designed for different clinical endpoints such as predicting the need for vasopressor support, intubation, ICU admission, or mortality [9,10,11]. In addition, widely utilized scoring systems for ICU settings, including Acute Physiology and Chronic Health Evaluation II (APACHE II) and Sequential Organ Failure Assessment (SOFA), were developed to assess organ dysfunction and predict prognosis across a wide spectrum of critically ill patients, regardless of the underlying etiology [12,13].

However, the predictive performance of currently used scoring systems in older adults with CAP who were admitted to the ICU remains unclear. While general ICU scores provide useful prognostic information across a broad range of critically ill patients, none of them were specifically developed for or validated in older adults with CAP [14,15,16]. Moreover, the discriminative power of PSI and CURB-65 for predicting mortality has been shown to decrease with advancing age [14]. Although SMART-COP has demonstrated the highest area under the curve (AUC) for predicting ICU admission and need for intubation in older adults with CAP, the study was not specifically designed for ICU patients but rather included all older adults with CAP [17].

To date, there is no validated scoring system specifically developed for older adults admitted to the ICU with CAP. Furthermore, none of the currently used pneumonia severity scores or general ICU scores have been adequately studied to predict adverse outcomes and mortality in this particular population. Therefore, the current study aimed to evaluate the predictive value of SMART-COP for adverse outcomes and mortality in older adults admitted to the ICU, a population that has been largely underrepresented in the existing literature.

## 2. Materials and Methods

### 2.1. Study Design

This prospective observational study was conducted at the ICU of the Ministry of Health Etlik City Hospital. A total of 227 consecutive patients aged 65 or older admitted to the ICU with a diagnosis of CAP between 1 December 2024 and 1 December 2025 were prospectively enrolled. CAP was defined as an acute infection of pulmonary parenchyma acquired outside of the hospital, characterized by compatible clinical features and the presence of new radiologic infiltrates, in accordance with the American Thoracic Society guideline [18]. Individuals were excluded if they had a different primary diagnosis, were not diagnosed as CAP at admission but developed pneumonia 48 h or more after hospital admission, were discharged with oral antibiotic therapy, were hospitalized in a general ward, were discharged from the hospital within 10 days, were admitted postoperatively, were under 65 years of age, or refused to participate in the study. SMART-COP and CURB-65 scores were calculated at hospital admission, whereas APACHE II and SOFA scores were calculated at the time of ICU admission. Written informed consent was obtained from all participants or their legal representatives.

### 2.2. Pneumonia Severity and ICU SCORES

#### 2.2.1. SMART-COP

SMART-COP is a severity assessment tool to predict intensive respiratory or vasopressor support in community-acquired pneumonia. It is composed of systolic blood pressure, multilobar chest radiography involvement, albumin level, respiratory rate, tachycardia, confusion, oxygenation (PaO_2_ or SpO_2_), and arterial pH, with age-adjusted cutoffs for patients aged ≤ 50 and >50 years. Total scores range from 0 to 11, with higher scores indicating greater disease severity, and are stratified into four risk categories: low (0–2), moderate (3–4), high (5–6), and very high (≥7) [11].

#### 2.2.2. CURB-65

CURB-65 is one of the most widely used tools for assessing CAP severity and predicting mortality risk. It incorporates confusion, urea, respiratory rate, blood pressure, and age (≥65 years). Scores range from 0 to 5, with higher values reflecting greater disease severity, and are stratified as low risk (0–1), intermediate risk (2), and high risk (≥3) [10].

#### 2.2.3. SOFA

SOFA was developed to evaluate the extent of organ dysfunction in critically ill patients. It evaluates six organ systems—respiratory, cardiovascular, hepatic, coagulation, renal, and neurological—with each component scored from 0 to 4 based on the degree of dysfunction. Total scores range from 0 to 24, with higher scores reflecting more severe organ dysfunction and a greater risk of mortality. A score of 2 or more in any individual organ system is generally considered to indicate organ dysfunction [12].

#### 2.2.4. APACHE II

APACHE II is a scoring system designed to assess disease severity and predict mortality in critically ill patients admitted to the ICU, regardless of the underlying diagnosis. It is composed of 12 acute physiological variables, age, and chronic health status, with the score calculated using the worst values recorded within the first 24 h of ICU admission. Total scores range from 0 to 71, with higher values indicating greater disease severity and increased predicted mortality [13].

### 2.3. Outcomes and Definitions

In this study, mortality refers to in-ICU mortality; accordingly, all patients discharged alive from the ICU, whether to a general ward or to hospice, were classified as survivors. The need for vasopressor support was defined as any dose of dopamine, epinephrine, or norepinephrine used to maintain the mean arterial pressure above 65 mmHg [19]. Hospital-acquired pneumonia was defined as pneumonia developing 48 h or more after hospital admission or being present at admission in people who had been discharged from the hospital within the last 7 to 10 days [20].

### 2.4. Data Collection

Data were collected prospectively using a standardized case report form during the ICU stay. In addition to severity scores, baseline demographic characteristics and comorbidities were recorded. Data on the history of long-term oxygen therapy, non-invasive mechanical ventilation (NIV) use, gastrostomy or tracheostomy, and previous hospitalizations were collected. We also obtained laboratory data including complete blood count, acute phase reactants, and blood urea nitrogen at admission. Variables pertaining to the ICU course were documented as well, including the type of respiratory support, need for intubation, successful extubation, development of hospital-acquired pneumonia (HAP) or ventilator-associated pneumonia (VAP), respiratory tract microbiology results, length of stay, and in-hospital mortality status.

### 2.5. Statistical Analysis

Statistical analyses were performed using IBM SPSS Statistics, version 27.0 (IBM Corp., Armonk, NY, USA), and R, version 4.5.1 (R Foundation for Statistical Computing, Vienna, Austria). The normality of continuous variables was assessed before analysis. Normally distributed variables are presented as mean ± standard deviation and non-normally distributed variables as median (minimum–maximum); categorical variables are presented as frequencies and percentages. Continuous variables were compared between survivors and non-survivors using the independent-samples t-test or the Mann–Whitney U test, depending on the distribution of the data, and categorical variables using the Pearson chi-square test or the Fisher exact test when expected cell counts were insufficient.

The discriminative ability of the SMART-COP, CURB-65, SOFA, and APACHE II scores for predicting four outcomes—in-ICU mortality, need for intubation, vasopressor requirement, and development of hospital-acquired pneumonia—was assessed using receiver operating characteristic (ROC) curve analysis. For each score and outcome, the area under the ROC curve (AUC) was reported with its 95% confidence interval, and the optimal cut-off was determined from the ROC coordinates as the point maximizing the Youden index (J = sensitivity + specificity − 1); the corresponding sensitivity, specificity, and positive and negative predictive values were calculated. For each outcome, the AUCs of the four scores—derived from the same patients—were compared pairwise (SMART-COP versus each of CURB-65, SOFA, and APACHE II) using the DeLong test for correlated ROC curves, performed with the pROC package in R; the AUC difference, its standard error, the z statistic, the *p* value, and the 95% confidence interval were reported. Given the exploratory nature of these pairwise comparisons and the limited number of prespecified hypotheses, no correction for multiple testing was applied; accordingly, the results—particularly those with borderline significance—are interpreted as hypothesis-generating rather than confirmatory. The association between length of ICU stay and each score was assessed using the Spearman rank correlation coefficient. A two-tailed *p* value < 0.05 was considered statistically significant.

### 2.6. Ethical Approval

The study protocol was conducted in compliance with the Declaration of Helsinki and was approved by the ethics committee of the Ministry of Health, Etlik City Hospital (AEŞH-BADEK-2024-971).

## 3. Results

The mean age of the 227 patients included in the study was 77.9 ± 8.7 years, and 131 (57.7%) were male. The most common comorbidities were hypertension (HT, 59.5%), chronic obstructive pulmonary disease (COPD, 34.8%), diabetes mellitus (DM, 33.9%), and congestive heart failure (CHF, 24.7%). At baseline, 31.3% of the patients were receiving long-term oxygen therapy (LTOT), 11.9% were on home NIV, 4.4% had a tracheostomy, and 7.5% had a gastrostomy (Table 1).

During the ICU stay, respiratory support was escalated as needed and, in some patients, had already been initiated before ICU admission: 19.4% received high-flow oxygen therapy, 41.4% non-invasive ventilation, and 52.9% invasive mechanical ventilation. Because support was often escalated in a stepwise manner, these categories are not mutually exclusive. Of the patients who received invasive mechanical ventilation, 22.5% were successfully weaned and extubated. Vasopressor support was required in 50.2% of the cohort. Bacterial growth in at least one respiratory tract sample was detected in 24.7% of the cohort, with *Streptococcus pneumoniae* (41.1%) being the most common bacteria, followed by *Haemophilus influenzae* and *Pseudomonas aeruginosa* (16.1% each). Viral growth was identified in 16.7% of the population, most commonly SARS-CoV-2 (39.5%), influenza A (23.7%), and rhinovirus (18.4%). Hospital-acquired pneumonia was diagnosed in 21.1% of the patients, and *Klebsiella* species (31.2%), *Acinetobacter* species (25.0%), and *P. aeruginosa* (22.9%) were the most commonly isolated microorganisms (Table 2). Overall, 56.8% of the patients were discharged to a general ward, and 4.0% to hospice, whereas 39.2% died in the ICU.

According to SMART-COP, 2.2% of the cohort were classified as low risk, 21.1% as moderate risk, 31.3% as high risk, and 45.4% as very high risk. SMART-COP category was significantly associated with mortality (*p* < 0.001): the proportion of patients classified as very high risk was higher among non-survivors (82.0%) than among survivors (21.7%). The mean SMART-COP score was also higher in non-survivors (7.54 ± 1.36) than in survivors (5.22 ± 1.81; *p* < 0.001), as were SOFA, CURB-65, and APACHE II scores (all *p* < 0.001) (Table 1). All four scores were higher in patients who required intubation than in those who did not, and likewise in patients who required vasopressor support than in those who did not (all *p* < 0.001) (Table 3). SMART-COP, SOFA, CURB-65, and APACHE II scores were all higher in patients who developed hospital-acquired pneumonia than in those who did not (*p* < 0.001 for SMART-COP, SOFA, and APACHE II; *p* = 0.002 for CURB-65). Length of ICU stay correlated weakly but significantly with CURB-65 (rho = 0.157, *p* = 0.018) and APACHE II scores (rho = 0.143, *p* = 0.031), whereas no significant correlation was found with SMART-COP or SOFA scores (*p* > 0.05).

**Figure 1:**
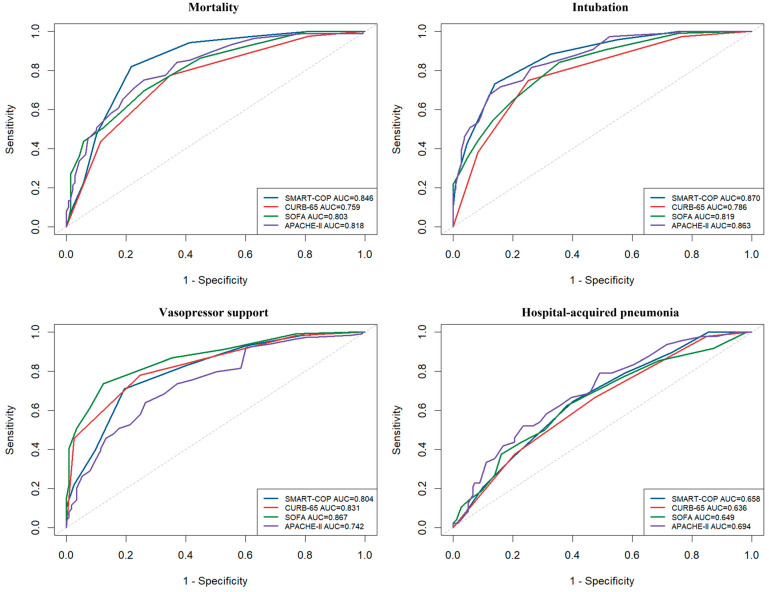
Comparison of ROC curves of scores according to outcomes. The dashed diagonal line represents the reference line of no discrimination (AUC = 0.5).

All four scores significantly predicted each of the four outcomes—mortality, intubation, vasopressor requirement, and the development of hospital-acquired pneumonia (HAP) (all *p* ≤ 0.001; Table 4, Figure 1).

For mortality, SMART-COP had the highest AUC (0.846, 95% CI 0.796–0.896); at a cut-off of 7, it achieved 82.0% sensitivity, 78.3% specificity, a PPV of 70.9%, and an NPV of 87.1%. It was followed by APACHE II (0.818), SOFA (0.803), and CURB-65 (0.759). SMART-COP showed the highest AUC for intubation as well (0.870, 95% CI 0.825–0.914); at a cut-off of 7, sensitivity was 73.3% and specificity 86.0% (PPV 85.4%, NPV 74.2%), with APACHE II (0.863), SOFA (0.819), and CURB-65 (0.786) close behind. For vasopressor requirement, SOFA performed best (AUC 0.867, 95% CI 0.822–0.913; cut-off 6: sensitivity 73.7%, specificity 87.6%, PPV 85.7%, NPV 76.7%), ahead of CURB-65 (0.831), SMART-COP (0.804), and APACHE II (0.742). Discrimination for HAP was modest across all scores; APACHE II had the highest AUC (0.694, 95% CI 0.613–0.775), but at a cut-off of 21, its specificity (50.8%) and PPV (30.2%) were low despite a high NPV (90.1%).

Pairwise comparison of the ROC curves refined this picture (Table 5). For mortality, SMART-COP discriminated significantly better than CURB-65 (ΔAUC 0.087, *p* = 0.003) but performed comparably to SOFA (ΔAUC 0.043, *p* = 0.186) and APACHE II (ΔAUC 0.028, *p* = 0.359). The same pattern held for intubation: SMART-COP outperformed CURB-65 (ΔAUC 0.084, *p* = 0.002), with no significant difference from SOFA (*p* = 0.091) or APACHE II (*p* = 0.800). For vasopressor requirement, SOFA discriminated significantly better than SMART-COP (ΔAUC 0.063, *p* = 0.039), while SMART-COP in turn outperformed APACHE II (ΔAUC 0.062, *p* = 0.045) and was comparable to CURB-65 (*p* = 0.337). No significant differences were found between any of the scores for HAP (all *p* > 0.38).

## 4. Discussion

Beyond being the most common infectious cause of hospital admission and death, CAP carries a substantial economic burden, as it frequently leads to ICU admission, prolonged length of stay, the need for intubation, and vasopressor support. Considering the aging of the population and the fact that increasing age is itself a risk factor for adverse outcomes, the demand for ICU care is likely to continue rising. Predicting the risk of adverse events and death allows clinicians both to manage patients optimally and to allocate ICU resources rationally. While more comprehensive data generally allow more accurate predictions, daily practice—particularly in the emergency department, where time is constrained—calls for tools that are quick and easy to apply. In our study, SMART-COP provided the highest discrimination for mortality and intubation and significantly outperformed CURB-65 for both, while performing at least as well as the general ICU scores SOFA and APACHE II. SOFA was the strongest predictor of vasopressor requirement, and none of the scores discriminated well for HAP.

While advanced age brings with it more comorbidities, more medications, and higher risks for patients, it also makes the disease harder to manage for clinicians [21]. Optimal management of pneumonia depends on determining the appropriate level of care early, so that treatment is matched to severity while limited resources—particularly ICU beds—are not used unnecessarily. CURB-65, a simple and widely used CAP tool, can help clinicians decide on the level of care, including ICU admission, but it was not designed to predict outcomes within the ICU [10]. SOFA and APACHE II, by contrast, are well-established tools for general ICU assessment, but their complexity limits their use at the bedside [12,13]. Against this background, SMART-COP—a simple, pneumonia-specific score calculable at first contact—matched the discrimination of the more complex ICU scores for mortality and intubation, the two outcomes most directly reflecting the need for organ support. Because it can be calculated quickly at the point of initial assessment from clinical and routine laboratory parameters, SMART-COP offers intensivists a practical tool for rapid risk stratification [11,17]. Moreover, because SMART-COP is derived from data available at hospital admission, whereas SOFA and APACHE II require parameters recorded at ICU admission—a mean of 27.7 ± 4.6 h later in our cohort—it identified high-risk patients earlier in the clinical course while achieving comparable discrimination. This lead time may have practical value. Recognizing high-risk patients at admission, before the deterioration that leads to ICU transfer, could allow clinicians to arrange closer monitoring or a higher level of care earlier, to prepare for intubation or vasopressor support rather than respond to it once it occurs, and to optimize antibiotic treatment sooner. Whether acting on this earlier window changes patient outcomes cannot be answered by our observational data and would need to be tested in interventional studies; still, a score that identifies these patients a mean of nearly 28 h before ICU admission provides a realistic opportunity for earlier intervention. It should be acknowledged, however, that SMART-COP was originally derived to predict the need for intensive respiratory or vasopressor support; its strong performance for intubation and vasopressor requirement therefore partly reflects the outcomes it was designed to capture. Its comparable discrimination for mortality—an outcome not explicitly embedded in its derivation—is thus particularly noteworthy.

When the underlying disorder is infection, vasopressor support is frequently required as sepsis progresses to septic shock, which is itself defined by a vasopressor requirement to maintain a mean arterial pressure of ≥65 mmHg together with hyperlactatemia [19]. Given that SOFA quantifies organ dysfunction and hemodynamic instability directly—its cardiovascular component is scored from mean arterial pressure and the dose of vasopressor administered—it is unsurprising that SOFA predicted vasopressor requirement better than the other scores [12]. This structural advantage should, however, be interpreted with caution: because the cardiovascular component incorporates vasopressor use itself, the association is at least partly built into the score. Notably, the limitations of the original cardiovascular component in reflecting contemporary vasopressor practice were among the factors that prompted a comprehensive revision of the score, which was published as SOFA-2 in 2025 [22]. More broadly, because each score captures different aspects of critical illness, the most appropriate tool may depend on the specific outcome of interest.

Neither SMART-COP nor the other scores performed well in predicting the development of hospital-acquired pneumonia, with all AUCs below 0.70 and no significant differences between scores. This is not unexpected: these tools were designed to capture the severity of the acute infection at presentation, whereas HAP develops later in the ICU course and is driven largely by factors unrelated to initial illness severity, such as the presence and duration of intubation, length of exposure, invasive devices, and prior antimicrobial exposure [20]. In older patients, this limitation may be compounded by age-related factors that these scores do not capture: susceptibility to HAP may be driven as much by frailty, immunosenescence, and colonization with resistant organisms as by the severity of the initial pneumonia. Predicting HAP in this population may therefore require dedicated models that incorporate these age-related and time-dependent factors rather than severity scores designed to quantify the acute physiological burden at presentation—an avenue worth exploring in future studies. Similarly, although CURB-65 and APACHE II showed statistically significant correlations with length of ICU stay, these were weak (rho < 0.16) and of negligible clinical relevance, while SMART-COP and SOFA showed no significant correlation. Length of stay is influenced by numerous factors beyond admission severity, including complications, comorbidity burden, and institutional discharge practices, and is therefore unlikely to be reliably predicted by any single severity score [23]. Taken together, these findings delineate the boundaries of these scores: they are prognostic instruments for early mortality and acute organ support, not general-purpose predictors of the entire ICU trajectory.

In our cohort, comorbidity burden was not associated with mortality in the way an established risk factor would predict. Most disorders common in older adults showed no significant relationship with mortality, and COPD was unexpectedly more frequent among survivors than non-survivors. Rather than indicating a protective effect, this likely reflects selection for ICU admission: patients with known COPD may be recognized and admitted earlier, at a less advanced stage of acute illness, than those without a prior respiratory diagnosis [24]. Laboratory markers were more informative. Consistent with previous reports, hypoalbuminemia was associated with increased mortality, as were higher BNP levels [25,26].

This study has several strengths. To our knowledge, it is among the few studies to compare a pneumonia-specific score (SMART-COP) directly against general ICU scores (SOFA, APACHE II) and CURB-65 across four distinct outcomes—mortality, intubation, vasopressor requirement, and hospital-acquired pneumonia—in an exclusively older ICU population with community-acquired pneumonia. The use of pairwise DeLong comparisons, rather than reliance on point estimates alone, allowed the differences between scores to be assessed statistically rather than assumed.

Several limitations should be acknowledged. First, this was a single-center study, which limits generalizability. Second, because we assessed in-ICU mortality, patients discharged to hospice were classified as survivors despite their likely poor short-term prognosis; this may have led to a degree of misclassification, and longer-term mortality was not captured. Third, although SMART-COP showed the numerically highest discrimination for mortality and intubation, its area under the curve did not differ significantly from those of SOFA or APACHE II, and the confidence intervals overlapped; the advantage over CURB-65 should therefore not be overstated as overall superiority. In addition, pairwise AUC comparisons were performed across four outcomes without adjustment for multiple testing; given the exploratory nature of these analyses, the significant differences observed—particularly the borderline values—should be interpreted with caution. Fourth, microbiological yield was low, and the etiological data should be interpreted with caution, as they may reflect prior antimicrobial exposure. Fifth, the scores were calculated at different time points—SMART-COP at hospital admission and SOFA and APACHE II at ICU admission, which occurred a mean of 27.7 h later. This interval reflects the way these scores are used in practice, but it also means that the comparison was not strictly contemporaneous: diagnostic and therapeutic interventions initiated before ICU admission may have influenced the physiological variables that determine the ICU scores. For example, some patients had already been started on respiratory support, such as high-flow oxygen therapy or non-invasive ventilation, before ICU admission, which may have modified the oxygenation and physiological parameters contributing to the SOFA and APACHE II scores. The admission-based and ICU-based scores should therefore be regarded as complementary, capturing risk at different stages of care, rather than as strictly interchangeable measures assessed under identical conditions. Finally, we used the original SOFA score; a revised version (SOFA-2) was published in 2025, and its performance in this population remains to be evaluated.

In older adults admitted to the ICU with community-acquired pneumonia, all four scores significantly predicted mortality, intubation, and vasopressor requirement, but none reliably predicted the development of hospital-acquired pneumonia. Among the scores evaluated, SMART-COP offered the best balance of simplicity and performance, matching the complex ICU scores for mortality and intubation while requiring only parameters available at hospital admission. Given that it can be calculated rapidly from clinical and routine parameters available at hospital admission, SMART-COP may serve as a practical early tool for risk stratification in this population. The most appropriate score, however, depends on the outcome of interest—SOFA was the strongest predictor of vasopressor requirement. Because this was a single-center study, its findings may reflect local case-mix and practice patterns; larger, multicenter studies across different healthcare settings are therefore needed to confirm the external validity of these results, and of SMART-COP in particular, before they can be generalized.

## Figures and Tables

**Table 1:** Baseline characteristics of patients with community-acquired pneumonia admitted to the ICU, and comparison between survivors and non-survivors.

Characteristic	Survivors (*n* = 138)	Non-Survivors (*n* = 89)	Total (N = 227)	*p*
Age, years, mean ± SD	77.43 ± 8.50	78.64 ± 8.97	77.91 ± 8.69	0.308
Male sex, *n* (%)	77 (55.8)	54 (60.7)	131 (57.7)	0.468
Time from hospital arrival to ICU admission (hours)	27.5 ± 4.6	28.0 ± 4.7	27.7 ± 4.6	0.420
**Comorbidities, *n* (%)**				
Hypertension	88 (63.8)	47 (52.8)	135 (59.5)	0.101
COPD	57 (41.3)	22 (24.7)	79 (34.8)	**0.010**
Diabetes mellitus	52 (37.7)	25 (28.1)	77 (33.9)	0.136
Congestive heart failure	30 (21.7)	26 (29.2)	56 (24.7)	0.264
Coronary artery disease	30 (21.7)	23 (25.8)	53 (23.3)	0.580
Malignancy	28 (20.3)	21 (23.6)	49 (21.6)	0.670
Dementia	22 (15.9)	13 (14.6)	35 (15.4)	0.933
Cerebrovascular disease	18 (13.0)	10 (11.2)	28 (12.3)	0.843
Chronic kidney disease	12 (8.7)	14 (15.7)	26 (11.5)	0.158
Parkinson disease	6 (4.3)	3 (3.4)	9 (4.0)	1.000
**Pre-existing organ support/devices, n (%)**				
Long-term oxygen therapy	44 (31.9)	27 (30.3)	71 (31.3)	0.806
Home non-invasive ventilation	17 (12.3)	10 (11.2)	27 (11.9)	0.971
Pre-existing gastrostomy	13 (9.4)	4 (4.5)	17 (7.5)	0.154
Pre-existing tracheostomy	4 (2.9)	6 (6.7)	10 (4.4)	0.235
**Admission laboratory values**				
White blood cells, ×10^9^/L, median (min–max)	12.80 (1.15–46.90)	14.10 (1.92–66.00)	13.60 (1.15–66.00)	**0.015**
Hemoglobin, g/dL, mean ± SD	11.03 ± 2.25	10.64 ± 2.35	10.87 ± 2.29	0.216
Neutrophils, ×10^9^/L, median (min–max)	10.25 (0.30–44.00)	13.10 (0.30–54.00)	11.40 (0.30–54.00)	**0.011**
Lymphocytes, ×10^9^/L, median (min–max)	0.78 (0.08–7.50)	0.65 (0.12–4.57)	0.72 (0.08–7.50)	0.052
Platelets, ×10^9^/L, mean ± SD	243.98 ± 111.67	195.52 ± 112.08	224.98 ± 114.08	**0.002**
Albumin, g/L, mean ± SD	31.43 ± 5.61	28.13 ± 5.50	30.14 ± 5.79	**<0.001**
BNP, pg/mL, mean ± SD †	5825.96 ± 8103.50	11,289.45 ± 12,363.93	7906.09 ± 10,265.74	**<0.001**
C-reactive protein, mg/L, median (min–max)	116.50 (8.00–1074.00)	133.00 (20.10–471.00)	128.00 (8.00–1074.00)	0.340
Procalcitonin, µg/L, median (min–max)	0.46 (0.03–100.00)	2.30 (0.03–100.00)	0.78 (0.03–100.00)	**<0.001**
**Severity scores, mean ± SD**				
SMART-COP	5.22 ± 1.81	7.54 ± 1.36	6.13 ± 2.00	**<0.001**
CURB-65	3.27 ± 0.92	4.19 ± 0.84	3.63 ± 0.99	**<0.001**
SOFA	4.55 ± 2.38	7.98 ± 3.28	5.89 ± 3.23	**<0.001**
APACHE II	19.51 ± 6.65	29.06 ± 8.47	23.26 ± 8.75	**<0.001**
**SMART-COP risk category, n (%)**				
Low risk	5 (3.6)	0 (0.0)	5 (2.2)	**<0.001**
Moderate risk	46 (33.3)	2 (2.2)	48 (21.1)	
High risk	57 (41.3)	14 (15.7)	71 (31.3)	
Very high risk	30 (21.7)	73 (82.0)	103 (45.4)	

† BNP was available for 218 patients. COPD, chronic obstructive pulmonary disease; BNP, B-type natriuretic peptide; SOFA, Sequential Organ Failure Assessment; APACHE II, Acute Physiology and Chronic Health Evaluation II. Statistically significant *p*-values are shown in bold.

**Table 2:** Microbiological findings.

Microorganism	*n*	%
**Bacterial pathogens (*n* = 56 with growth)**		
*Streptococcus pneumoniae*	23	41.1
*Haemophilus influenzae*	9	16.1
*Pseudomonas aeruginosa*	9	16.1
Methicillin-susceptible *S. aureus*	8	14.3
*Klebsiella* spp.	7	12.5
*Acinetobacter* spp.	1	1.8
**Viral pathogens (*n* = 38 with detection)**		
SARS-CoV-2	15	39.5
Influenza A	9	23.7
Rhinovirus	7	18.4
Adenovirus	4	10.5
Seasonal coronavirus	2	5.3
Respiratory syncytial virus	1	2.6
**Hospital-acquired pneumonia pathogens (*n* = 48)**		
*Klebsiella* spp.	15	31.2
*Acinetobacter* spp.	12	25.0
*Pseudomonas aeruginosa*	11	22.9
*Stenotrophomonas* spp.	4	8.3
*Corynebacterium* spp.	3	6.2
Methicillin-resistant *S. aureus*	2	4.2
*Serratia* spp.	1	2.1

Percentages for bacterial and viral pathogens are calculated among patients with a positive respiratory culture (*n* = 56, one patient had two isolates) or viral detection (*n* = 38), respectively; HAP pathogen percentages are calculated among the 48 patients who developed hospital-acquired pneumonia. spp., species; SARS-CoV-2, severe acute respiratory syndrome coronavirus 2.

**Table 3:** Severity scores according to mortality, intubation, vasopressor requirement, and development of hospital-acquired pneumonia.

Outcome	Score	Absent, Mean ± SD	Present, Mean ± SD	*p*
Mortality	SMART-COP	5.22 ± 1.81	7.54 ± 1.36	**<0.001**
	SOFA	4.55 ± 2.38	7.98 ± 3.28	**<0.001**
	CURB-65	3.27 ± 0.92	4.19 ± 0.84	**<0.001**
	APACHE II	19.51 ± 6.65	29.06 ± 8.47	**<0.001**
Intubation	SMART-COP	4.79 ± 1.56	7.33 ± 1.53	**<0.001**
	SOFA	4.08 ± 2.07	7.51 ± 3.22	**<0.001**
	CURB-65	3.09 ± 0.87	4.11 ± 0.84	**<0.001**
	APACHE II	17.70 ± 5.48	28.21 ± 8.13	**<0.001**
Vasopressor requirement	SMART-COP	5.07 ± 1.72	7.18 ± 1.68	**<0.001**
	SOFA	3.89 ± 1.72	7.88 ± 3.15	**<0.001**
	CURB-65	3.04 ± 0.77	4.22 ± 0.83	**<0.001**
	APACHE II	19.65 ± 7.10	26.82 ± 8.79	**<0.001**
Hospital-acquired pneumonia	SMART-COP	5.89 ± 2.00	7.00 ± 1.73	**<0.001**
	SOFA	5.52 ± 3.02	7.29 ± 3.61	**<0.001**
	CURB-65	3.53 ± 1.00	4.02 ± 0.89	**0.002**
	APACHE II	22.01 ± 8.32	27.92 ± 8.83	**<0.001**

Values are mean ± SD. “Absent” and “Present” refer to the presence of each outcome. SOFA, Sequential Organ Failure Assessment; APACHE II, Acute Physiology and Chronic Health Evaluation II. Statistically significant *p*-values are shown in bold.

**Table 4:** Discriminative performance of the four scores for predicting each outcome.

Outcome	Score	AUC	95% CI	Cut-Off	Sens. (%)	Spec. (%)	PPV (%)	NPV (%)
Mortality	SMART-COP	0.846	0.796–0.896	7	82.0	78.3	70.9	87.1
	APACHE II	0.818	0.763–0.873	23	75.3	73.9	65.0	82.3
	SOFA	0.803	0.747–0.860	6	69.7	73.9	63.3	79.1
	CURB-65	0.759	0.699–0.819	4	77.5	65.2	59.0	81.8
Intubation	SMART-COP	0.870	0.825–0.914	7	73.3	86.0	85.4	74.2
	APACHE II	0.863	0.817–0.909	23	71.7	84.1	83.5	72.6
	SOFA	0.819	0.767–0.872	5	84.2	64.5	72.7	78.4
	CURB-65	0.786	0.729–0.842	4	75.0	74.8	76.9	72.7
Vasopressor requirement	SOFA	0.867	0.822–0.913	6	73.7	87.6	85.7	76.7
	CURB-65	0.831	0.782–0.880	4	78.1	75.2	76.1	77.3
	SMART-COP	0.804	0.748–0.860	7	71.1	80.5	78.6	73.4
	APACHE II	0.742	0.678–0.806	23	64.0	73.5	70.9	66.9
Hospital-acquired pneumonia	APACHE II	0.694	0.613–0.775	21	79.2	50.8	30.2	90.1
	SMART-COP	0.658	0.577–0.739	7	64.6	59.8	30.1	86.3
	SOFA	0.649	0.560–0.737	6	62.5	62.0	30.6	86.0
	CURB-65	0.636	0.555–0.717	4	66.7	52.5	27.4	85.5

Scores are ordered by AUC within each outcome. AUC, area under the ROC curve; CI, confidence interval; Sens., sensitivity; Spec., specificity; PPV, positive predictive value; NPV, negative predictive value.

**Table 5:** Pairwise comparison of the area under the ROC curve between SMART-COP and each comparator score (DeLong test).

Outcome	Comparison	AUC SMART-COP	AUC Comparator	ΔAUC	z	*p*
Mortality	SMART-COP vs. CURB-65	0.846	0.759	0.087	2.943	**0.003**
	SMART-COP vs. SOFA	0.846	0.803	0.043	1.322	0.186
	SMART-COP vs. APACHE II	0.846	0.818	0.028	0.917	0.359
Intubation	SMART-COP vs. CURB-65	0.870	0.786	0.084	3.050	**0.002**
	SMART-COP vs. SOFA	0.870	0.819	0.050	1.692	0.091
	SMART-COP vs. APACHE II	0.870	0.863	0.007	0.253	0.800
Vasopressor requirement	SMART-COP vs. CURB-65	0.804	0.831	−0.027	−0.961	0.337
	SMART-COP vs. SOFA	0.804	0.867	−0.063	−2.060	**0.039**
	SMART-COP vs. APACHE II	0.804	0.742	0.062	2.001	**0.045**
Hospital-acquired pneumonia	SMART-COP vs. CURB-65	0.658	0.636	0.022	0.545	0.586
	SMART-COP vs. SOFA	0.658	0.649	0.009	0.200	0.841
	SMART-COP vs. APACHE II	0.658	0.694	−0.036	−0.872	0.383

ΔAUC is the AUC of SMART-COP minus that of the comparator; a positive value favors SMART-COP. Bold *p* values denote a statistically significant difference (*p* < 0.05). AUC, area under the ROC curve. Statistically significant *p*-values are shown in bold.

## Data Availability

The data presented in this study are available on request from the corresponding author. The data are not publicly available due to privacy restrictions.

## Abbreviations

The following abbreviations are used in this manuscript:

CAP	Community-acquired pneumonia
ICU	Intensive care unit
PSI	Pneumonia Severity Index
APACHE II	Acute Physiology and Chronic Health Evaluation II
SOFA	Sequential Organ Failure Assessment
NIV	Non-invasive mechanical ventilation
HAP	Hospital-acquired pneumonia
VAP	Ventilator-associated pneumonia
ROC	Receiver operating characteristic
AUC	Area under the ROC curve
COPD	Chronic obstructive pulmonary disease
HT	Hypertension
DM	Diabetes mellitus
CHF	Congestive heart failure
LTOT	Long-term oxygen therapy
BNP	B-type natriuretic peptide
SARS-CoV-2	Severe acute respiratory syndrome coronavirus 2
PPV	Positive predictive value
NPV	Negative predictive value
